# Targeting the PDGF signaling pathway in tumor treatment

**DOI:** 10.1186/1478-811X-11-97

**Published:** 2013-12-20

**Authors:** Carl-Henrik Heldin

**Affiliations:** 1 Ludwig Institute for Cancer Research, Science for life laboratory, Uppsala University, Box 595SE-751 24 Uppsala, Sweden

## Abstract

Platelet-derived growth factor (PDGF) isoforms and PDGF receptors have important functions in the regulation of growth and survival of certain cell types during embryonal development and *e.g.* tissue repair in the adult. Overactivity of PDGF receptor signaling, by overexpression or mutational events, may drive tumor cell growth. In addition, pericytes of the vasculature and fibroblasts and myofibroblasts of the stroma of solid tumors express PDGF receptors, and PDGF stimulation of such cells promotes tumorigenesis. Inhibition of PDGF receptor signaling has proven to useful for the treatment of patients with certain rare tumors. Whether treatment with PDGF/PDGF receptor antagonists will be beneficial for more common malignancies is the subject for ongoing studies.

## Introduction

Platelet-derived growth factor (PDGF) isoforms stimulate growth, survival and motility of mesenchymal cells and certain other cell types [1,2]. They have important functions during embryonal development and in the control of tissue homeostasis in the adult. Overactivity of PDGF signaling is associated with the development of certain malignant diseases, as well as non-malignant diseases characterized by excessive cell proliferation. The involvement of PDGF overactivity in non-malignant diseases has been discussed in a recent review [3]. The present review will focus on the role of PDGF signaling in tumor development, and on the use of PDGF antagonists in tumor treatment.

### PDGF isoforms

**Figure 1:**
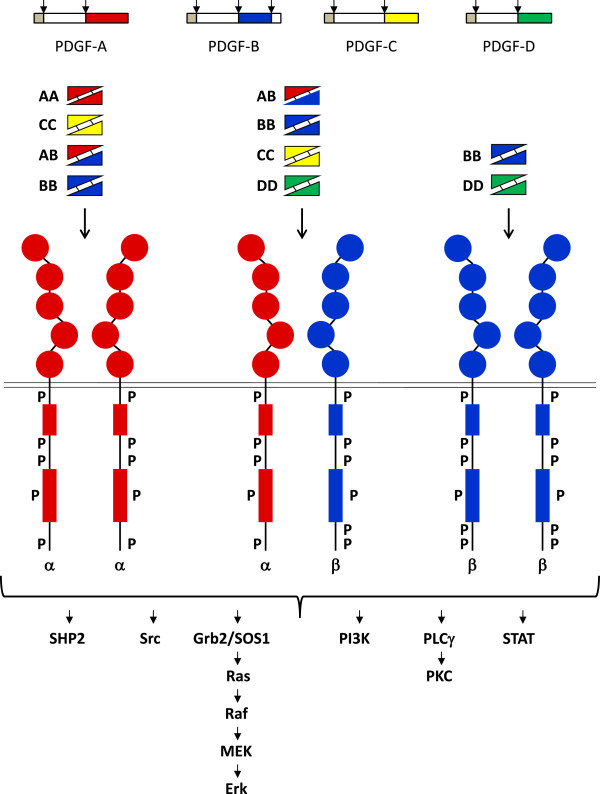
**Binding of the five PDGF isoforms induces different homo- and heterodimeric complexes of PDGFRα and PDGFRβ.** The PDGF isoforms are synthesized as precursor molecules with signal sequences (grey), precursor sequences (open) and growth factor domains (red, blue, yellow and green). After dimerization, the isoforms are proteolytically processed (arrows) to their active forms which bind to the receptors. The extracellular parts of the receptors contain 5 Ig-like domains; ligand binding occurs preferentially to domains 2 and 3, and domain 4 stabilizes the dimer by a direct receptor-receptor interaction. The intracellular parts of the receptors contain tyrosine kinase domains split into two parts by an intervening sequence. Ligand-induced dimerization induces autophosphorylation of the receptors, which activates their kinases and create docking sites for SH2-domain-containing signaling molecules, some of which are indicated in the figure. Activation of these signaling pathways promotes cell growth, survival, migration and actin reorganization.

The PDGF family consists of disulphide-bonded homodimers of A-, B-, C- and D-polypeptide chains, and the heterodimer PDGF-AB. The PDGF isoforms are synthesized as precursor molecules. PDGF-AA, -AB and –BB are cleaved already inside the producer cells in secretory vesicles. In contrast, PDGF-CC and –DD are secreted as inactive precursor molecules; N-terminal CUB-domains need to be cleaved off to activate the growth factors. This cleavage serves an important regulatory role, and is performed by tissue-type plasminogen activator (tPA) or plasmin in the case of PDGF-CC, and by urokinase-type PA (uPA) or matriptase (MT-Sp1) in the case of PDGF-DD [4-7] (Figure 1).

### Signaling via PDGF receptors

**Figure 2:**
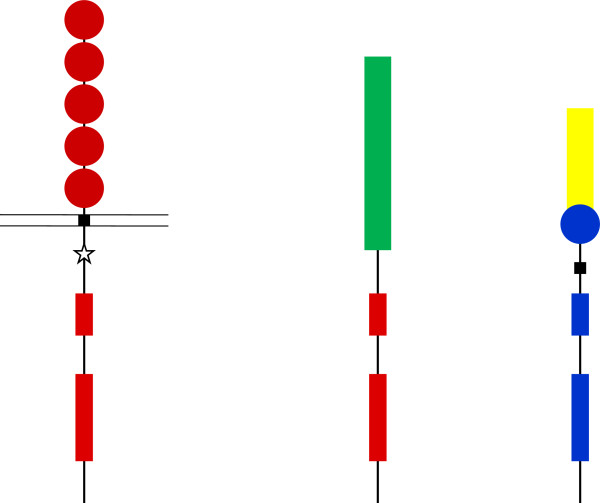
**Mutation of PDGF receptors in malignancies.** PDGFRα (left part) has been found to be activated by point mutations in about 5% of GIST cases. In the figure a mutation is indicated (star) in the juxtamembrane domain, but can occur also in other parts of the protein. In HES the intracellular part of PDGFRα (red) has been found to be fused to FIP1L1 (green), and in CMML the intracellular part of PDGFRβ (blue) has been found to be fused to TEL (yellow). Other fusion partners have also been identified.

PDGF isoforms exert their cellular effects by binding to α- and β-tyrosine kinase receptors (PDGFRα and PDGFRβ, respectively). The two PDGF receptors are structurally similar and consist of extracellular domains with five immunoglobulin (Ig) - like domains and intracellular parts with kinase domains which contain characteristic inserts of about 100 amino acid residues without homology to kinases. Ligand binding occurs mainly to Ig-like domains 2 and 3, and causes dimerization of the receptors, which is further stabilized by direct receptor-receptor interactions involving Ig-like domain 4 [8,9]. The dimerization is a key event in activation since it brings the intracellular parts of the receptors close to each other promoting autophosphorylation *in trans* between the receptors. The PDGF polypeptide chains bind to the receptors with different affinities. Thus, PDGF-AA, -AB, -BB and -CC induce αα receptor homodimers, PDGF-BB and PDGF-DD ββ receptor homodimers, and PDGF-AB, -BB, -CC and –DD αβ receptor heterodimers Figure 1; [2].

The autophosphorylation serves two important functions. First, it changes the conformation of the intracellular part of the receptor so that the kinase is activated. There is no 3-dimensional structure yet for PDGF receptors, so precise information about mechanisms that control the kinase is not available. However, it is likely that in the resting state, the kinase is kept inactive by at least three mechanisms: *i*) The activation loop in the kinase domain is likely to be folded over the catalytic cleft; autophosphorylation of a conserved tyrosine residue in this region causes the loop to move away from the active site [10]. *ii*) The juxtamembrane part of the receptor is likely to be folded in a loop which restricts the access to the active site; autophosphorylation of two tyrosine residues in this region changes the conformation and enhances the kinase activity [11]. *iii*) The C-terminal tail of the receptor is most likely folded over the kinase domain; autophosphorylation of two C-terminally located tyrosine residues relieves the kinase of this inhibition [12]. Similar regulatory mechanisms have been observed in the structurally related colony stimulating factor-1 receptor (CSF1R) and FLT3.

Second, autophosphorylation creates docking sites for SH2-domain-containing signaling molecules. The α- and β-receptors contain 10 and 11 known autophosphorylated tyrosine residues, respectively [13]. About 10 different families of SH2-domain-containing molecules have been shown to selectively bind to different phosphorylated residues in the PDGF receptors. These include signaling molecules with intrinsic enzymatic activities, such as tyrosine kinases of the Src family, the SHP-2 tyrosine phosphatase, phospholipase C-γ (PLC-γ) and the GTPase activating protein (GAP) for Ras. Moreover, the receptors bind and activate signal transducers and activators of transcription (STATs), which after activation are translocated to the nucleus where they act as transcription factors. Finally, the receptors bind adaptor molecules which lack intrinsic enzymatic activities, but can form complexes with other signaling molecules. Examples include the regulatory subunit p85 of the phosphatidylinositol 3′-kinase (PI3K), which forms complex with the p110 catalytic subunit, and Grb2 which binds the nucleotide exchange molecule SOS1, activating Ras and the Erk MAP-kinase pathway (Figure 1). In addition, the PDGF receptors bind other adaptors, *e.g.* Shc, Nck, Crk and GAB, which mediate interactions with a plethora of different downstream signaling molecules. The activation of these signaling pathways leads to cell proliferation and survival, as well as to actin reorganization and cell migration. The extensive cross-talk between the different signaling pathways makes it difficult to assign individual pathways to specific responses; in a cell-type- and context-dependent manner, several signaling pathways contribute to each of the cellular responses.

### Modulation and termination of PDGF receptor signaling

Signaling via PDGF receptors is carefully controlled and modulated. In the early phase of signaling different mechanisms assure that the signal rapidly reaches sufficient strength. For instance, in PDGF stimulated cells reactive oxygen species are produced in a PI3-kinase-dependent pathway, which inhibit tyrosine phosphatases by reacting with a cysteine residue in their active site [14,15]. Another mechanism that amplifies the signaling is the ubiquitination and degradation of MAP-kinase phosphatase 3, which dephosphorylates and inactivates Erk MAP-kinase; removal of this phosphatase enhances Erk MAP-kinase activation [16].

There are also mechanisms that negatively modulate PDGF signaling. One example is the docking of Ras-GAP to the activated PDGFRβ; this counteracts the activation of Ras which occurs by the simultaneous docking of the Grb2-SOS1 complex [17]. Interestingly, PDGFRα does not bind Ras-GAP and therefore activates Erk MAP-kinase more efficiently than PDGFRβ [18].

Negative modulatory effects are also exerted by tyrosine phosphatases which dephosphorylate and inactivate PDGF receptors. Examples of such phosphatases include PTP1B [19], TC-PTP [20] and PTPRJ/DEP-1 [21,22]. In addition, the tyrosine phosphatase SHP-2 binds to PDGF receptors and dephosphorylates the receptors and their substrates. However, SHP-2 also positively modulates signaling, *e.g.* via dephosphorylation of a C-terminal inhibitory phosphorylation site in Src family members, thereby activating them [23], or by acting as an adaptor for binding of the Grb2-SOS1 complex, thus promoting Ras activation [24].

Other mechanisms of modulation of PDGF receptor signaling are exerted by interactions with other cell surface receptors. Thus, PDGF receptor interaction with other tyrosine kinase receptors, such as the EGF receptor [25], has been observed. Moreover, PDGF receptors have been shown to interact with non-kinase receptors; thus, integrins [26] and the low density lipoprotein receptor-related protein [27-29] enhance signaling, whereas interaction with the hyaluronan receptor CD44 suppresses signaling [30].

Activation of PDGF receptors triggers internalization of the receptors in a clathrin- and dynamin-dependent manner. Internalization is promoted by ubiquitination of the receptors by the ubiquitin ligase Cbl [31]. Signaling continues in endosomes [32] until the receptors are degraded in proteasomes and lysosomes. Alternatively, receptors can be sorted to recycling vesicles whereby they reappear at the plasma membrane where they can signal again. One mechanism which promotes sorting of receptors to recycling is exerted by activation of PLCγ and the downstream protein kinase C (PKC) [33], another involves PI3-kinase-mediated uptake of the receptor via an alternative internalization route, *i.e.* macropinocytosis [34]. Increased receptor recycling is accompanied by an increased amplitude and duration of signaling.

### Normal function of PDGF isoforms and receptors

The physiological functions of PDGF have been analyzed using mice with the genes for PDGF isoforms or receptors knocked-out. These studies have elucidated important roles for PDGF isoforms in the development of mesenchymal cell types of different organs reviewed in [2]. Often PDGF isoforms are produced by epithelial or endothelial cells and act in a paracrine manner on nearby mesenchymal cells, such as fibroblasts, pericytes and smooth muscle cells. Thus, signaling via PDGFRα is important for the development of the facial skeleton, hair follicles, spermatogenesis oligodendrocytes and astrocytes [35], as well as for the development of the lung [36] and intestinal villi [37]. Signaling via PDGFRβ is important for the development of blood vessels, kidneys [38-41] and white adipocytes [42].

In the adult, PDGF stimulates wound healing [43]. It also regulates the intestinal fluid pressure in tissues and thereby counteracts edema formation [44].

### PDGF signaling antagonists

The involvement of PDGF overactivity in malignant diseases (see further below), as well as certain non-malignant diseases [3], has led to the development of different types of antagonists of PDGF signaling that now are under preclinical and clinical evaluation.

The developed inhibitors include antibodies, DNA aptamers or soluble extracellular parts of the receptors that bind PDGF isoforms and thus prevent their binding to signaling receptors [45,46]. Alternatively, antibodies or other binders can target the receptors and prevent their activation or promote their degradation [47-49]. These types of antagonists have the advantage of being reasonably specific, however, they are expensive and cumbersome to administer. Another type of antagonists are low molecular inhibitors of the receptor kinases (Table 1). Several potent inhibitors of PDGF receptor kinases have been developed, including imatinib, sunitinib, sorafenib, pazopanib and nilotinib. None of these inhibitors are specific; they all have their characteristic profiles of inhibition of different other kinases. Thus, imatinib, in addition to inhibiting PDGF receptor kinases, inhibits the stem cell receptor (Kit) and Abl kinases, and sunitinib inhibits vascular endothelial cell growth factor (VEGF) receptors and Flt3; sorafenib has an inhibitory profile similar to sunitinib, but also inhibits the serine/threonine kinase Raf. Although the lack of specificity contributes to side effects and can be seen as a disadvantage, experience has shown that it is often advantageous to hit more than one kinase in tumor treatment.

**Table 1:** Characteristics of PDGF receptor kinase inhibitors

**Inhibitor**	**Primary targets**	**Secondary targets**
Imatinib	Abl, **PDGFR**, Kit	Raf
Sunitinib	**PDGFR**, VEGFR, Kit, Flt3	
Sorafenib	Raf, VEGFR, **PDGFR**, Kit, Flt3	FGFR
Pazopanib	VEGFR, **PDGFR**, Kit	FGFR
Nilotinib	Kit, Abl, **PDGFR**	
Cediranib	VEGFR, Kit, **PDGFR**	FGFR
Motesanib	VEGFR, Kit	**PDGFR**, Ret
Axitinib	VEGFR	**PDGFR**, Kit
Linifenib	VEGFR, Kit	**PDGFR**
Dasatinib	Abl, Src	**PDGFR**, Kit
Quizartinib	FLT3	Kit, **PDGFR**, Ret, CSF1R
Ponatinib	Ret, Abl	**PDGFR**, VEGFR

The table summarizes the specificities of some kinase inhibitors targeting PDGF receptors. The K_d_:s of the different members of the PDGFR, VEGFR and FGFR families are often similar and are lumped together, for simplicity. As primary targets are listed the kinases that are inhibited at the lowest concentrations (regardless of absolute concentrations). As secondary targets are listed kinases that are inhibited by about 10-fold higher inhibitor concentrations. For references, see [3,50-54].

### PDGF signaling in malignant diseases

There are several observations supporting the notion that overactivity of PDGF signaling can drive tumorigenesis [55]. In certain tumors, PDGF or PDGF receptor genes are mutated, alternatively, their expressions are increased. Thus, in the rare skin tumor dermatofibrosarcoma protuberance (DFSP), the gene encoding PDGF-B is fused to the gene encoding collagen 1A1 [56,57]. This leads to the production of large amounts of a fusion protein consisting of N-terminal collagen sequence and C-terminal PDGF-B sequence. After processing, a PDGF-BB-like protein is released which stimulates the growth and survival of the producer fibroblasts in an autocrine manner [58].

The PDGF receptor genes have also been found to be mutated in certain malignancies. Point mutations in the PDGFRα gene occur in about 5% of gastrointestinal stromal tumors (GIST); these mutations lead to amino acid residue replacements in critical regions of the receptor causing activation of the kinase [59]. In GIST, similar mutations in the structurally related receptor Kit, is even more common. PDGF receptor genes have been found involved in gene rearrangements in certain leukemias [60]. Thus, the intracellular parts of both PDGFRα and PDGFβ genes have been found to be fused to different partner genes that encode molecules that can oligomerize; the combination of loss of regulatory sequences in the juxtamembrane and transmembrane parts of the receptors and their oligomerization activate the receptor kinases. Moreover, in 5-10% of glioblastoma multiforme cases, the α-receptor gene is amplified resulting in expression of a high number of receptors [61-63]. Amplification of PDGFRα has also been observed in oligodendrogliomas [64], esophageal squamous cell carcinoma [65], and artery intimal sarcomas [66,67]. This makes the cells susceptible to stimulation by lowered amounts of PDGF, or if the number of receptors become high enough, signaling may occur in a PDGF-independent manner. An activating deletion mutation in the PDGFRα gene has also been detected in a human glioblastoma [68].

During tumorigenesis, epithelial tumors may undergo epithelial-mesenchymal transition (EMT), which is associated with increased invasiveness and metastasis [69]. During EMT, PDGF receptor expression by the tumor cells increases, so that epithelial tumors that initially did not respond to PDGF may become responsive to PDGF stimulation [70]. The expression of PDGF isoforms are also part of the EMT program, which may enhance PDGF receptor signaling by autocrine stimulation.

PDGF produced by tumor cells or non-tumorigenic cells, such as endothelial cells and macrophages, also acts on non-tumor cells in solid tumors. Thus, pericytes around blood vessels, and fibroblasts and myofibroblasts in the stroma, carry PDGF receptors and respond to PDGF. Pericytes are dependent on PDGF produced by endothelial cells and have an important role during angiogenesis [40]. PDGF stimulation of fibroblasts and myofibroblasts in the stroma contributes to the increased interstitial fluid pressure (IFP) in solid tumors. The increased IFP is an obstacle in chemotherapeutic treatment of tumors, since it decreases the transcapillar flow and decreases drug uptake reviewed by [71].

The fact that PDGF receptor signaling is often overactive in tumors has prompted attempts to treat patients with various malignancies with PDGF/PDGF receptor antagonists. During tumor progression, tumor cells acquire a number of mutations, some of which drive tumorigenesis. It has been observed that tumor cells often become “addicted” to the signaling pathways that are activated by mutational events, and that inhibition of such pathways induces apoptosis of the tumor cells [72]. On the other hand, after some time re-growth of the tumor often occurs, due to the appearance of various types of resistance mechanisms. The involvement of PDGF signaling in specific tumor types and the possible usefulness of PDGF antagonists in tumor treatment (Table 2), are discussed in the following sections. First, mechanisms operating in the tumor cells themselves are discussed; the involvement of PDGF stimulation in the stroma compartment is discussed in a later chapter.

**Table 2:** Use of PDGFR kinase inhibitors in clinical trials for different tumors

**Tumor type**	**Results of patient studies**	**Refs**
Glioblastoma multifome	Only limited effects of single agent treatment by imatinib in Phase II and Phase III studies.	[103,104]
	No significant effect of imatinib treatment in combination with hydroxyurea.	[105,106,108]
Chordoma	1 PR and 35 SD out of 50 patients treated, were observed in a Phase II study.	[110]
Meningeoma	No or only modest effect of imatinib as single agent or combined with hydroxyurea.	[111,112]
	Among 9 patients preselected for PDGFR expression, 7 SD were noted.	[113]
Dermatofibrosarcoma protuberance	In a Phase II study, 4 CR and 4 PR out of 12 patients treated were recorded.	[126]
	In other Phase II trials, PR was noticed in about half of the patients.	[117,127-130]
Gastrointestinal stromal tumor	Imatinib and other tyrosine kinase inhibitors against PDGFRα and Kit are used routinely in the clinic with good results	[132-138]
Soft tissue sarcoma	In a Phase III study with 369 patients a median progression-free survival of 4.6 months was noted in patients treated with pazopanib compared with 1.6 months for untreated controls	[149]
Osteosarcoma	No advantage of treatment with imatinib as single agent.	[141]
	Some effect of imatinib in combination with everolimus in treatment of synovial sarcoma.	[142]
Chronic myeloproliferative diseases	Patients with CMML with rearrangement of PDGFRβ responded to imatinib.	[168]
Hypereosinophilic syndrome	Patients with HES responded to imatinib.	[169-172]
	Patients who developed resistance to imatinib responded to nilotinib or sorafenib.	[174,175]
Prostate cancer	Out of 44 patients with hormone-refractory prostate cancer treated with sunitinib, 1 had PR, 3 a decline in prostate specific antigen of >50%, and 9 had a significant improvement in pain.	[181]
	No increased survival upon treatment with imatinib.	[195,196]
Non-small cell lung cancer	Combination treatment with imatinib and docetaxel yielded 1 PR and 4 SD out of 20 treated patients.	[213]
	2 PR and 7 SD were observed after treatment of 18 patients with sunitinib.	[214]
Neuroblastoma	Little or no effect by imatinib as single agent treatment of children with relapsed or refractory neuroblastoma.	[236]

CR, complete response; PR, partial response; SD, stable disease.

### Brain tumors

A clear demonstration that autocrine stimulation by PDGF can drive the development of glioblastoma multiforme (GBM) was the finding that simian sarcoma virus (SSV) induces brain tumors in marmoset monkeys [73]; the transforming oncogene of SSV, v-*sis*, encodes a PDGF-B-like molecule [74,75]. In human material, increased expression of PDGF isoforms and PDGF receptors have been demonstrated in GBM cell lines [76,77] and in tumor tissue [78-84]. Notably, a malignancy-dependent increased expression was noticed where the α-receptor was primarily expressed in the tumor cells, and the β-receptor in the stromal cells. Amplification of the PDGF α-receptor has been demonstrated, but is not as common as amplification of the EGF receptor [61]. Mutations in the PDGFRα gene, in the parts encoding the extracellular as well as the intracellular domains [85-88], have been observed; in addition, a fusion with the VEGFR2 gene has been found [89].

The importance of autocrine stimulation by PDGF has been verified in animal models, in which a retrovirus encoding PDGF-B was injected in newborn mice [90,91]. PDGF-induced transformation was found to be enhanced by mutations in certain tumor suppressor genes, such as *Ink4/Arf*, *TP53* and *PTEN*[92-94]. In cells with Ink4a/Arf deficiency, PDGFRα promotes tumorigenesis via the SHP2/PI3K/Akt/mTOR pathway [95].

PDGF overexpression forces differentiation of glial cells to the oligodendrocyte lineage and promotes the development of highly malignant oligodendroglial tumors in mice [96-98]. The transforming efficiency of PDGF stimulation is illustrated by the fact that overexpression of PDGF-B in corpus callosum causes GBM also in adult rats [99]. Overexpression of the long isoforms of PDGF-A, which has a retention motif enhancing its autocrine stimulatory effect, was also found to efficiently promote GBM development [100].

Glioma stem cells preferentially express PDGFRβ and its activation promotes glioma stem cell self-renewal, suggesting that targeting of this receptor can be beneficial in treatment of glioma patients [101]. PDGF-B depletion completely abrogated the tumor initiating capacity of glioma stem cells [102].

Despite the finding that the PDGF receptor kinase inhibitor imatinib enhances the cytotoxicity of radiation in a mouse glioma model [103], only limited effects were recorded by imatinib treatment in Phase II clinical trials in glioblastoma patients [104,105]. Subsequent Phase II and Phase III studies explored the combination between imatinib and hydroxyurea in the treatment of recurrent glioblastoma, but no clinically meaningful antitumor effect was observed [106-108].

In addition to glioblastoma, PDGF overactivity has been implicated also in other types of brain tumors. PDGFRα and β have been shown to be overexpressed in ependymoma of children and expression of PDGFα was found to correlate to poor prognosis [109].

Chordoma is a rare slow-growing tumor arising from remnants of the notochord, which often expresses PDGFRβ. Following encouraging treatment results of occasional patients with imatinib [110], a Phase II clinical study was organized. Among 50 patients treated, one partial response and 35 patients with stable disease were recorded [111].

Meningeomas are mostly benign tumors with good prognosis that are treated with surgery, but some are inoperable and requires other treatment. Since meningiomas often express PDGF receptors, treatments of recurrent meningeomas with single-agent imatinib [112] or with imatinib plus hydroxyurea [113] have been tried; however, no or only modest effect was recorded. On the other hand, more encouraging results were obtained in a small study with preselected patients with recurrent meningeomas with expression of at least one of the PDGF receptors; whereas no complete or partial responses were seen, seven out of nine patients showed stable disease after imatinib treatment [114].

PDGF receptors, as well as c-Kit, have been found to be overexpressed and overactivated in peripheral and vestibular schwannomas [115]. Treatment of vestibular schwannoma cells [116] or other types of primary schwannoma cells [117], lacking the tumor suppressor NF2, with nilotinib inhibited the growth of the cells *in vitro*.

### Sarcomas

Like in the case of glioblastomas, the normal counterpart cells of sarcomas express PDGF receptors. Overexpression of PDGF isoforms may then stimulate cell growth and survival in autocrine and paracrine manners. The clearest example that such mechanisms can drive tumorigenesis is the rare skin tumor DFSP, which is characterized by a gene rearrangement placing the collagen 1A1 gene upstream of the PDGF-B gene [118]. This leads to the production of a fusion protein which is processed to a molecule similar to mature PDGF-BB and causes autocrine stimulation of growth [57,58,119].

Inhibition of PDGF receptor signaling by the kinase inhibitor imatinib inhibits the growth and promotes apoptosis of DFSP cells [120,121]. Treatment with imatinib has also shown beneficial effects for individual patients with DFSP [122-126]. These encouraging findings prompted a multicenter Phase II trial; out of 12 patients with DFSP, 4 showed complete and 4 partial responses [127]. The median time to progression was 24 months [128]. Additional Phase II trials showed partial responses in about half of the cases; however, the response were rather short-lived whereafter resistance mechanisms occurred [118,129-131].

In about 5% of patients with GIST, PDGFRα is activated by point mutations [59]. Treatment with imatinib has been shown to improve the outcome for GIST patients [132-134]. Upon development of resistance to imatinib, other kinase inhibitors, such as sunitinib [135] and nilotinib [136-138] have shown efficacy.

PDGF and PDGF receptors are also expressed in other types of sarcomas. Early studies revealed that a human osteosarcoma cell line, U-2OS, secretes a PDGF-like growth factor and shows autocrine receptor activation by this factor [139]. Malignancy-dependent co-expression of PDGF and PDGF receptors have also been observed in biopsies of soft tissue sarcoma [140,141], osteosarcoma [142] and synovial sarcoma [143]. Nearly all cases of Ewing’s sarcoma show the presence of the chimeric transcription factor EWS/ETS which causes upregulation of PDGF-C; treatment of a cell line from a Ewing’s sarcoma with a PDGFR kinase inhibitor was shown to inhibit its anchorage-independent growth [144].

Whereas treatment of osteosarcoma patients with imatinib did not show any advantage as a single agent [142], the combination of the mTOR inhibitor everolimus and imatinib may be useful in the treatment of synovial sarcoma [143].

PDGFRα is selectively upregulated in rhabdomyosarcoma [145,146], and PDGFRα expression is associated with poor prognosis [147,148]. Treatment with imatinib or a neutralizing PDGFRα antibody inhibited growth of alveolar rhabdomyosarcoma in a mouse model [146].

In a large randomized, double-blind, placebo-controlled Phase III trial, 369 patients with metastatic non-adipocytic soft tissue sarcoma who had failed on standard therapy, were subjected to treatment with pazopanib or not [149]. A median progression-free survival of 4.6 months and an overall survival of 12.5 months were recorded for the pazopanib treated patients, compared to 1.6 and 10.7 months, respectively for untreated patients. This study thus showed that treatment with pazopanib is of some advantage for these patients with sarcomas.

PDGF and PDGF receptors are also overexpressed in dog hemangiosarcoma, a malignant neoplasia of vascular endothelial cells [150]. Treatment of hemangiosarcoma in dogs with imatinib and dasatinib augmented the response to doxorubicin; however, dasatinib, which inhibits Src in addition to PDGF receptor kinases, was more efficient [151].

### Leukemias and lymphomas

Activating mutations in the *Abl* and *JAK2* genes, encoding tyrosine kinases, are common in myeloproliferative diseases; in some cases mutations are also seen in the *PDGFRα* and *PDGFRβ* genes [60].

In chronic monomyelocytic leukemia (CMML) the PDGFRβ gene has been found to be fused with the gene encoding the transcription factor TEL; the N-terminal of the fusion protein contains sequences from TEL which is followed by the intracellular part of the receptor containing the kinase domain [152] (Figure 2). There are also other fusion partners, including Rabaptin 5 [153], HIP1 [154] and H4 [155]. In a case of thrombocythemia, the tumor suppressor gene *KANK1* was found to be fused with the PDGFRβ gene [156].

PDGFRα are also rearranged in certain malignancies. Thus, in hypereosinophilic syndrome (HES), the α-receptor gene is fused to the FIP1L1 gene [157-159] (Figure 2). Activating point mutations in the PDGF α-receptor have also been seen [160].

These proteins all have the ability to oligomerize and thus cause clustering of the receptor kinase; the juxtaposition of the kinase domains, as well as the loss of inhibitory transmembrane [161] and juxtamembrane [162] sequences, cause activation of the kinase. Moreover, escape of ubiquitin-mediated degradation causing accumulation of large amounts of the fusion proteins may also contribute to the transformation [163].

Activation of the PDGFRβ kinase through gene rearrangements leads to chronic myeologenous leukemia (CML) or CMML [152], whereas activation of the PDGFRα kinase causes HES or chronic eosinophilic leukemia [164-166]. Interestingly, activation of yet other tyrosine kinases is associated with other types of leukemia, *i.e.* activation of the FLT3 kinase with acute myelocytic leukemia (AML) or myelodysplastic syndrome, and activation of the Kit kinase with aggressive mastocytosis, myelodysplastic syndrome and AML for references see [166]. It is likely that these differences reflect differences in the activation of signaling pathways. FIP1L1-PDGFRα activates STAT5, PI3-kinase and the Ras-Erk and p38 MAP-kinase pathways; particularly, the stronger activation of Erk and p38 MAP-kinases by FIP1L1-PDGFRα, compared to TEL-PDGFRβ, could be linked to eosinophilic differentiation [167]. Both TEL-PDGFRα and FIP1L1-PDGFRα fusion proteins activate the transcription factors STAT1, 3 and 5, and nuclear factor-κB (NFκB), and cause proliferation and differentiation towards the eosinophilic lineage [168].

Patients with CMML have been successfully treated with imatinib [169], as have patients with HES [170-173]. Point mutations conferring imatinib resistance have been described both for TEL-PDGFRβ [174] and FIP1L1-PDGFRα [157]. Other kinase inhibitors, such as nilotinib or sorafenib [175], or midostaurin (PKC412) [164], could inhibit some of the resistant kinase mutants, and offers alternative treatments [176]. Interestingly, only few cases of resistance due to point mutations in the PDGFR kinase domains have been reported, in contrast to the situation for the treatment of chronic myeloic leukemia in which such mutations in the kinase domain of Brc-Abl are very common; it has been suggested that this is because the PDGFRα kinase domain contains only few residues which can be exchanged resulting in interference with inhibitor binding, without loss of kinase activity [177].

Large granular lymphocyte leukemia results from the expansion of cytotoxic T cells or natural killer cells, cell types that have been shown to express PDGF receptors [178,179]. Together with stimulation by IL-15, autocrine stimulation by PDGF-BB drives the development of this rare leukemia, and a PDGF-BB neutralizing antibody was found to inhibit growth and survival of the leukemia cells [180].

Anaplastic large cell lymphoma is an aggressive non-Hodgkin’s lymphoma, which is characterized by the occurrence of a fusion between nucleophosmin and the tyrosine kinase ALK. In a mouse model of this disease, the AP-1 members Jun and JunB were found to promote the expression of PDGF-B in the lymphoma cells [181]. The importance of the autocrine PDGF stimulation for tumorigenesis is illustrated by the finding that treatment of the transgenic mice with imatinib significantly prolonged their life. Moreover, treatment of a patient with anaplastic large cell lymphoma with imatinib resulted in rapid, complete and sustained remission [181].

### Prostate cancer

Immunohistochemical stainings have revealed that PDGFRβ is upregulated in most primary and metastatic prostate cancer cells [182]. Moreover, PDGFRβ mRNA expression was identified by microarray analyses as one of five mRNAs that predict prostate cancer recurrence, the other four being chromogranin A, HOXC6, IPTR3 and sialyltransferase-1 [183]. Whereas the PDGFRβ ligand PDGF-B has not been found to be overexpressed in prostate tumors, the other PDGFRβ ligand, PDGF-D, is often expressed at high levels and its expression correlates to the degree of malignancy [7]. Overexpression of PDGF-D in a mouse model significantly enhanced prostate carcinoma onset and invasiveness [184]. Loss of PTEN, which enhances PI3-kinase signaling, promotes production of PDGF-D, whereas the AMP-activated kinase (AMPK) regulates PDGF-B expression [185]. Overexpression of PDGF-D in PC3 prostate cancer cells was found to promote EMT and a stem cell phenotype, which may explain the increased invasiveness [186]. When PDGF-B and PDGF-D were transfected into non-malignant prostate epithelial cells, PDGF-D was found to induce cell migration and invasion more efficiently than PDGF-B [187]. The stronger effect of PDGF-D was dependent of the Jun MAP-kinase and involved shedding and activation of the serine protease matriptase. The mechanism behind the stronger tumorigenic effect of PDGF-D, compared to PDGF-B that binds to the same receptor, remains to be elucidated.

Interestingly, PDGF-D, but not PDGF-B, was able to induce osteoclast differentiation, and to upregulate the expression and nuclear translocation of nuclear factor of activated T cells 1 (NFAT-1), a master regulator of osteoclastogenesis [188]. This production of PDGF-D by prostate cancer cells is likely to be of importance for the establishment of bone metastases.

PDGFRα has also been implicated in prostate cancer. In a preclinical model of disseminated prostate cancer, it was shown that treatment with a neutralizing antibody against PDGFRα inhibited the growth of skeletal metastases [189,190]. Moreover, knock-down of PDGFRα, as well as PDGFRβ, by siRNA suppressed growth of prostate cancer cells in mice and suppressed tumor angiogenesis [191]. Interestingly, evidence have been presented that a soluble component of the bone marrow can activate PDGFRα, and promote bone metastasis of prostate cancer cells, through a mechanism that does not require ligand-binding or receptor dimerization [192].

Preclinical studies have demonstrated potential benefit of inhibition of PDGFRβ signaling by imatinib in prostate cancer [193,194]. Whereas a Phase I clinical trial with imatinib combined with docetaxel showed some benefit [195], placebo-controlled clinical trials did not show any significantly increased progression free or overall survival [196,197]. Further clinical trials were halted because of excessive side effects; possibly, other PDGF receptor kinase inhibitors would be more useful. An interesting candidate is cediranib, which inhibits PDGF and VEGF receptor kinases and has been shown to inhibit intraosseous growth of PDGF-D positive prostate cancer cells in a mouse model [198].

### Liver cancer

During the progression of hepatocellular carcinoma, and in conjunction with epithelial-mesenchymal transition (EMT), PDGF-A as well as PDGFRα and β are induced [199]. Inhibition of PDGF receptor signaling was found to decrease cell migration *in vitro* and tumor growth *in vivo*, in a β-catenin-dependent manner, indicating an important role for PDGF signaling in hepatocyte tumor progression [200]. Sorafenib, which in addition to PDGFR inhibits Raf, VEGFR and Kit, is now standard treatment for patients with hepatocellular carcinoma. However, it is not clear how important PDGFR kinase inhibition is for the beneficial effects [201,202].

PDGF-A and PDGFRα mRNA and protein are often overexpressed in patients with cholangiocarcinoma; treatment of cholangiocarcinoma cell lines with the PDGF receptor kinase inhibitors imatinib or sunitinib suppressed cell viability and migration [203]. Sorafenib also inhibited cholangiocarcinoma cell growth and survival *in vitro* and *in vivo*[204]. In another study, myofibroblast-derived PDGF-BB was shown to provide survival signals for cholangiocarcinoma cells, thus protecting them from TRAIL-mediated cytotoxicity by enhancing Hedgehog signaling [205]. Targeting PDGFRα by imatinib sensitized cholangiocarcinoma cells to apoptotic stimuli *in vitro* and *in vivo*[206].

### Non-small cell lung cancer

PDGF receptors are not expressed, or expressed at low levels, in normal lung epithelial cells, however, expression of PDGFRα has been reported in lung cancer cell lines and tumor tissue [207-209]. Expression of PDGFRβ was also seen, but mainly in the stromal cells. Increased expression of PDGFRβ was seen in the rare sarcomatoid type of non-small cell lung cancer [210]. Expression of PDGF and PDGF receptors in lung cancer was found to be associated with poor prognosis [211]. Inhibition of PDGF in preclinical models of non-small cell lung cancer by treatment with a neutralizing PDGFRα antibody (MEDI-575) caused a significant decrease in stromal fibroblast content but had only minor effect on tumor cell proliferation [212]. In addition, transfection of a non-receptor binding mutant of PDGF-A (PDGF-0) in A549 lung cancer cells, which inactivates the PDGF produced by these cells, led to a markedly decreased tumor growth *in vivo* because of impaired recruitment of peri-endothelial cells [213].

A Phase II clinical study explored the effect of imatinib combined with docetaxel for the treatment of recurrent non-small cell lung cancer, however, only one partial response and 4 stable disease out of 23 treated patients were seen [214]; thus, the study did not reach its objective. Another study focused on Asian patients who were treated with sunitinib; two partial responses and 7 stable disease were observed out of 18 patients treated [215]. Larger studies need to be conducted before it is possible to determine whether inhibition of PDGF receptors, with or without inhibition of VEGF receptors, is of any benefit for lung cancer patients.

### Breast cancer

In breast cancer, expression of PDGF in tumor cells and PDGF receptors in stromal cells have been reported [216,217]. PDGF receptors are also expressed in the tumor cells, which correlate with tumor progression and invasion [70,218,219]. PDGF receptors have been observed to be upregulated upon IGF1 receptor independence in an animal model [220].

Combining imatinib treatment with radiotherapy showed a significantly stronger inhibition of cell proliferation compared to radiotherapy alone in a mouse model for breast cancer [221].

PDGF-D produced by cells in the stroma of breast cancers, *e.g.* adipose tissue-derived stem cells, was found to induce EMT of the cancer cells in a paracrine manner, thereby promoting the formation of cancer stem cells and tumorigenesis [222].

It remains to be determined whether inhibition of PDGF receptor signaling is of benefit for breast cancer patients. A possible subgroup that could benefit is patients with estrogen receptor positive tumors undergoing aromatase inhibition therapy, since this treatment has been found to be associated with an upregulation of PDGFRβ on the tumor cells [223].

### Colorectal cancer

In colorectal cancers PDGF receptors are mainly expressed by stromal cells and pericytes [224,225], but PDGF receptor expression has also been noted on colorectal carcinoma cell lines [226,227]. Expression of PDGF receptors is associated with poor prognosis for patients with colorectal cancer [228]. Studies using preclinical models have shown that colorectal cancer cells can acquire PDGFRβ in conjunction with EMT, and that activation of this receptor promotes metastasis [229].

### Other tumors

Several other tumor types have been reported to involve overactive PDGF signaling in the tumor cells. Thus, PDGF-D and PDGFRβ were found to be co-expressed in several mesothelioma cell lines, resulting in autocrine stimulation of cell proliferation [230].

In Wilms’ tumor of the kidney, PDGF-A and PDGFRα was expressed in 50% and 55% of the cases, respectively, in a cohort of 62 patients; interestingly, expression of PDGF-A and PDGFRα correlated with good prognosis [231]. It is possible that expression of PDGF-A and PDGFRα reflect a differentiated phenotype and therefore correlates to favorable prognosis. This is in contrast to breast [232], ovarian [233] and lung [207,234] carcinomas, in which cases PDGF and PDGF receptor expression correlate to poor prognosis.

The childhood tumor neuroblastoma arises from the neural crest remnants of the sympathetic nervous system, and has been shown to express PDGF receptors [235], as well as c-Kit [236]. Whereas imatinib inhibited neuroblastoma cells *in vitro* and in xenografts, little or no treatment effect as single agent was seen in children with relapsed or refractory neuroblastoma [237].

Activation of the hedgehog pathway occurs frequently in basal cell carcinoma of the skin. The transcription factor Gli1, which is activated in the hedgehog pathway, activates the promoter of the PDGFRα gene and thus promotes PDGFRα expression; this is an important mechanism by which hedgehog signaling promotes tumorigenesis [238], and suggests that PDGF inhibition could be beneficial in skin tumor treatment.

Leydig cell tumors of the testis express high levels of PDGF isoforms and PDGF receptors [239]. However, treatment of a patient with imatinib was not successful [240]. On the other hand, human testicular germ tumors also express PDGF receptors, and treatment with sunitinib as single agent showed beneficial effects even in cisplatin-resistant tumors in a mouse model [241].

Overexpression of PDGF-A, -B and –C isoforms and both PDGF receptors were found to be crucial for the development of thyroid nodules and recurrent goitre [242].

### Targeting PDGF in tumor stroma

In addition to tumor cells, non-tumor cells in solid tumors, such as macrophages and endothelial cells, produce PDGF isoforms. PDGF receptors are expressed on pericytes and smooth muscle cells of vessels, as well as on fibroblasts and myofibroblasts. Recent studies have shown that targeting of cells in tumor stroma can be beneficial in tumor treatment, particularly if combined with targeting of the tumor cells directly.

### Anti-angiogenic treatment

Angiogenesis is promoted by several different factors, including VEGF, FGF, TGFβ, angiopoietins and PDGF [243]. A monoclonal antibody against VEGF, bevacizumab, is already used clinically. PDGF has an accessory role in angiogenesis and, in particular, promotes pericyte recruitment to vessels. Studies using different mouse models have shown that anti-angiogenic therapy can be more efficient by combination of inhibition of VEGF signaling, targeting endothelial cells, and PDGF signaling, targeting pericytes [243-248]. Combination therapy probably interrupts the trophic relationship between endothelial cells and pericytes. Simultaneous inhibition of fibroblast growth factor (FGF) may be even more beneficial [50,249]. Interestingly, resistance to anti-VEGF treatment has been shown to involve increased expression of PDGF-C [250]. However, the effect of anti-PDGF treatment may be context-dependent. Thus, no synergistic effect was seen by the combination of anti-VEGF and anti-PDGF treatment in mouse models of colorectal and pancreatic cancer; in fact PDGF overexpression was found to inhibit endothelial cells and angiogenesis by intensive pericyte recruitment [251]. Another complication was reported from a clinical study in which CDP860, an engineered Fab’ fragment inhibiting PDGFRβ, was used; the study had to be interrupted since seven of eight patients developed fluid retention and three significant ascites upon treatment [47].

Bone-marrow-derived mesenchymal stem cells have been shown to exert an anti-angiogenic effect in preclinical models of glioma by inhibiting the recruitment of endothelial progenitor cells through decreased expression of PDGF-BB and other angiogenic factors [252].

A mechanism whereby PDGF-BB promotes tumor angiogenesis and tumor growth was recently presented; by induction of erythropoietin, PDGF-BB promotes endothelial cell proliferation, migration, sprouting and tube formation, and promotes extramodullary hematopoiesis leading to increased oxygen perfusion and protection against tumor-associated anemia [253]. Another mechanism was unraveled by studies of chronic lymphocytic leukemia; PDGF secreted by these tumor cells stimulated mesenchymal stromal cells to produce VEGF [254].

PDGF-BB has also been shown to stimulate lymphangiogenesis [255], and to promote lymphatic metastasis in gastric carcinoma [256]. In papillary thyroid cancer, expression of PDGFRα correlated with lymphatic metastases [257].

### Cancer-associated fibroblasts

It has become increasingly appreciated that stromal cells of solid tumors contribute to tumorigenesis [258,259]. Such cells include, in addition to vascular cells, *e.g.* macrophages and cancer-associated fibroblasts (CAFs). The latter cell type is heterogeneous and may derive from tissue fibroblasts, bone-marrow-derived progenitor cells or transdifferentiating epithelial cells. The various cell types of the stromal compartment contribute to tumorigenesis by secreting various growth factors and cytokines which promotes growth, survival and migration of the tumor cells, as well as epithelial-mesenchymal transition and tumor angiogenesis.

PDGF receptors are expressed on CAFs and there are several reports that PDGF stimulation affects CAF function. Thus, ectopic expression of PDGF-BB was found to promote stroma formation and tumor growth of melanoma [260], tumorigenesis of immortalized keratinocytes [261] and growth of prostate cancer [262]. Tumor cell-derived PDGF-AA was found to recruit CAFs in xenograft studies of breast [263] and lung [264] carcinomas. Transgenic expression of PDGF-CC in mouse liver cells resulted in tissue fibrosis and promoted development of hepatocellular carcinoma [265]. Moreover, expression of PDGF-CC in mouse models promoted recruitment of CAFs and growth of malignant melanoma [266] and liver metastasis of colorectal cancer [267]. Finally, ectopic expression of PDGF-DD was found to promote tumorigenesis and angiogenesis [268,269]. Stromal PDGF receptor expression has been shown to be associated with poor prognosis in breast and prostate cancer [270,271], in colorectal cancer [228,272] and in pancreatic carcinoma [273].

CAFs and myofibroblasts make contacts with collagen fibers of the extracellular matrix. PDGF stimulation of these cells causes cell contraction leading to an increased tumor interstitial pressure [71]. This is an obstacle in treatment of tumor patients with chemotherapy, since it decreases transcapillary transport and drug uptake. Treatment of mice with different types of solid tumors with PDGF antagonists was found to decrease IFP, to increase drug uptake, and to improve the efficiency of treatment with chemotherapeutic drugs [274-276]. In addition, treatment with VEGF antagonists was also found to decrease tumor IFP [277,278], and the combination of PDGF and VEGF antagonists gave an additive effect [279].

Targeting PDGF receptors in the stroma has been found to inhibit lung cancer growth [280,281] and bone metastasis [282], and colon cancer growth and metastasis [283] in mouse models.

### Future perspectives

The fact that PDGF and/or PDGF receptors are overexpressed or mutated in different tumors makes it desirable to investigate whether PDGF or PDGF receptor antagonists can be used to treat patients with these diseases. Some encouraging results have already been obtained by treatment of some rather rare tumors driven by overactive PDGF receptor signaling due to mutations of either PDGF or PDGF receptor genes. However, resistance mechanisms limit the success of such treatments, and anti-PDGF receptor treatment most likely will have to be combined with other signal transduction inhibitors, chemotherapeutical agents or other treatments, in order to achieve long lasting remissions.

In solid tumors PDGF receptors are expressed on pericytes of vessels and on fibroblasts and myofibroblasts of the stroma. Tumor cells are dependent on their environment for their proliferation and survival, making non-malignant PDGF receptor expressing cells interesting targets in tumor treatment. Further studies are needed in order to explore whether anti-PDGF receptor treatment targeting non-malignant cells in the tumor, in combination with anti-tumor cell treatment, will be of benefit for patients. It also remains to be determined whether selective inhibition of PDGF or PDGF receptors by *e.g.* monoclonal antibodies or ligand traps, or more unspecific inhibition of PDGF receptor kinases by low molecular weight inhibitors, will give the best clinical results.

## Competing interest

The author declare that he has no competing interests.
